# Associations of Dietary Antioxidants with Glycated Hemoglobin and Insulin Sensitivity in Adults with and without Type 1 Diabetes

**DOI:** 10.1155/2022/4747573

**Published:** 2022-06-25

**Authors:** Arpita Basu, Amy C. Alman, Janet K. Snell-Bergeon

**Affiliations:** ^1^Department of Kinesiology and Nutrition Sciences, School of Integrated Health Sciences, University of Nevada Las Vegas, USA; ^2^College of Public Health, University of South Florida, Tampa, FL, USA; ^3^Barbara Davis Center for Childhood Diabetes, University of Colorado, Anschutz Medical Campus, USA

## Abstract

Type 1 diabetes (T1D) has been associated with increased risks of atherosclerotic cardiovascular disease, and poor glycemic control and oxidative stress play a major role in its pathology. There is a lack of data on the role of dietary antioxidant micronutrients, including vitamins and trace elements, in glycemic control in T1D. The aim of this study is to examine associations of dietary intakes of micronutrients with glycemic status. We report data from a cross-sectional analysis from the coronary artery calcification in type 1 diabetes (CACTI) study (*n* = 1257; T1D: *n* = 568; nondiabetic controls: *n* = 689) collected between the years 2000 and 2002. Participants completed a validated food frequency questionnaire, a physical examination, and biochemical analyses. Linear regression was used to examine the associations of dietary antioxidant micronutrients with HbA1c and estimated insulin sensitivity (eIS) in models adjusted for relevant covariates and stratified by diabetes status. In adults with T1D, we observed higher dietary manganese intake associated with higher eIS in the model adjusted for age, sex, diabetes duration, and total calories. In nondiabetic controls, higher intake of manganese associated with lower HbA1c and higher eIS values that persisted in models adjusted for all relevant covariates. On the other hand, dietary copper revealed a positive association with HbA1c in models adjusted for all covariates, except BMI and plasma lipids. No associations were noted for vitamins C and E and dietary carotenoids in either group. These findings reveal dietary antioxidant micronutrients, especially trace elements such as copper and manganese deserve special attention in glycemic control in adults with T1D as well as in nondiabetic controls.This trial is register with NCT00005754.

## 1. Introduction

Type 1 diabetes (T1D) has been associated with a significant burden of atherosclerotic cardiovascular disease (ASCVD) and subsequent mortality when compared to the general population [1, 2]. Oxidative stress plays a key role in the initiation and development of ASCVD [3], and diabetes itself has been shown to aggravate oxidative stress in a plethora of mechanistic studies [4, 5]. Thus, dietary antioxidants can counteract and inhibit the adverse effects of oxidative stress, especially in adults with T1D. Dietary recommendations by the American Diabetes Association focus on dietary patterns that are high in natural antioxidants derived from fruits, vegetables, whole grains, and dairy [6]. While dietary micronutrients, such as vitamins C and E, manganese, and carotenoids are potent antioxidants [7–9], dietary iron and copper as redox metals can exacerbate oxidative stress and promote vascular damage and pancreatic dysfunction [10]. Furthermore, despite the antioxidant activities of vitamins C and E, review reports show no overall benefits of their supplementation in adults with type 2 diabetes [11]. To our knowledge, there are few reports on the habitual consumption of dietary antioxidant vitamins and minerals/trace elements, and their associations with glycemic control in adults with T1D as well as in the general population.

Cross-sectional studies reveal intakes of antioxidant vitamins, especially vitamins C and E that do not meet the dietary recommendations in adults with T1D; however, these studies do not report their associations with glycemic control [12, 13]. Using data from the US National Health and Nutrition Examination Survey (NHANES) in a cross-sectional study of approximately 24,000 adults, significant inverse associations of dietary patterns rich in vitamins, trace elements, and fiber, with insulin resistance or glycemic control, were observed in adults with and without any form of diabetes [14]. While epidemiological data show an association, clinical trials are often conflicting and mostly show null results of vitamin supplementation on CVD and diabetes outcomes in large trials [15, 16]. While most of the studies focus on antioxidant vitamins C and E, few reports are available on the role of trace elements/minerals involved in oxidative stress pathways, such as dietary iron and copper. Emerging data show a positive association of dietary iron intake and diabetes risks. In a meta-analysis of 11 prospective studies, Bao et al. reported a significant positive association of heme iron intake with type 2 diabetes, as well as with body ferritin stores [17]. In a smaller narrative review of only four studies, dietary iron intake was significantly associated with increased risks of T1D [18]. These data on micronutrients of antioxidant activities are important in supporting effective dietary recommendations in adults with T1D as well as for those at increased risk for ASCVD. In addition to glycated hemoglobin (HbA1c), the estimated insulin sensitivity (eIS), a model based on waist circumference, triglycerides, adiponectin, and diastolic blood pressure, is a validated model to examine insulin sensitivity in T1D [19]. However, no studies have examined the associations of antioxidant nutrient intakes with eIS in adults with T1D which leads to the scope of the current report.

The Coronary Artery Calcification in Type 1 Diabetes (CACTI) is a prospective study examining the progression of ASCVD and its association with dietary, lifestyle, and biochemical factors in adults with T1D compared to matched nondiabetic controls. We have previously reported altered macronutrient intakes, especially higher dietary fats from animal sources [20], and dietary patterns characterized by convenience foods and baked desserts to be positively associated with HbA1c in adults with T1D [21]. Using dietary data from the same cohort, we now aim to examine the associations of dietary intakes of trace elements (iron, copper, zinc, selenium, and manganese) and vitamins (vitamins C and E and carotenoids) that regulate antioxidant/oxidative stress pathways, with HbA1c and eIS in adults with and without T1D. We aim to examine data at the baseline time point in models adjusted for relevant covariates including dietary patterns and food groups.

## 2. Methods

### 2.1. Study Design and Participants

Data presented in this report were collected at baseline of the CACTI study as previously reported [20]. All study participants provided informed consent, and the study protocol was approved by the Colorado Multiple Institutional Review Board (ethics code 97-661). The study was registered at clinicaltrials.gov (NCT00005754).

### 2.2. Dietary Data and Glycemic Control

Detailed dietary data were obtained using a food frequency questionnaire (Harvard 1988) as previously described [20]. Baseline data collection took place between March 2000 and April 2002. Participants fasted overnight (12 h) for blood draws to determine blood lipids (LDL-cholesterol and triglycerides) and HbA1c. Anthropometric measurements included body weight, height, and waist circumference, as well as systolic blood pressure (SBP) and fifth-phase diastolic blood pressure (DBP) measured during the rest state and an average of three measurements being reported. As an index of insulin sensitivity, eIS was calculated based on a method validated by Duca et al. using a best-fit prediction model based on waist circumference, triglycerides, adiponectin, and diastolic blood pressure [19].

### 2.3. Statistical Analyses

Differences in baseline characteristics were analyzed between adults with and without T1D using Student *t* test for continuous variables and *χ*^2^ test for categorical risk factors. In addition, Wilcoxon rank sum test was used to analyze variables with a skewed distribution. Factor analysis was used to examine dietary patterns, and foods were considered to form part of the dietary patterns with a load factor greater than 0.4. A multiple linear regression model was used to examine associations of dietary antioxidant micronutrients with HbA1c and eIS as clinical markers of glycemic control. Models were adjusted for relevant covariates as follows: model 1 (age, sex, total calories, and diabetes duration for T1D), model 2 (model 1 + BMI), model 3 (model 1 + plasma LDL-cholesterol and triglycerides), and model 4 (model 1 + dietary patterns and food groups). These models were stratified by diabetes as we observed significant interaction effects. All *P* values were 2-tailed, and main effects and interaction effects were considered if *P* was ≤0.05. Analyses were performed using SAS version 9.4 (SAS Institute Inc., Cary, NC).

## 3. Results

### 3.1. Baseline Characteristics

Adults with T1D were younger in age and had a greater proportion of females than the nondiabetic controls. As expected, T1D adults had higher serum HbA1c and lower eIS than the nondiabetic controls. Lipid profiles were lower in the T1D group (LDL-cholesterol and triglycerides). No significant differences were noted in BMI and total caloric intake between the two groups. Among the dietary intake of antioxidant micronutrients, vitamins C and E, as well as iron, copper, zinc, selenium, manganese, and carotenoids, did not differ between the two groups (Table 1).

### 3.2. Dietary Antioxidants and HbA1c

In a multivariate model adjusting for relevant covariates including dietary patterns and food groups, no associations were observed between any of the dietary antioxidant micronutrients and serum HbA1c in adults with T1D. On the other hand, in nondiabetic controls, dietary copper revealed a significant positive association with HbA1c in models adjusted for age, sex, total calories, dietary patterns, and food groups. We also observed a significant inverse association of dietary manganese intake with HbA1c in nondiabetic controls persisting in all adjusted models (Table 2).

### 3.3. Dietary Antioxidants and Insulin Sensitivity (eIS)

In adults with T1D, dietary intake of manganese revealed a significant positive association with insulin sensitivity only in the minimally adjusted model. On the other hand, in nondiabetic controls, dietary manganese intake was consistently associated with higher insulin sensitivity in all adjusted models. Finally, we also observed a borderline significant inverse association of dietary copper intake with insulin sensitivity in non-diabetic controls (Table 3).

## 4. Discussion

Overall, this cross-sectional analysis shows a positive association of dietary manganese with insulin sensitivity that remained significant in all adjusted models in adults without diabetes and remained significant in minimally adjusted model in T1D group. We also observed higher intake of copper associated with higher HbA1c levels in nondiabetic controls, thus indicating poor glycemic control; this association remained significant in models adjusted for all dietary patterns as well as age, sex, and total calories. Finally, we also observed a consistent protective (inverse) association of the trace element manganese with HbA1c that remained significant in all adjusted models in nondiabetic controls.

Dietary copper is a redox active metal and has been shown to cause impaired glucose metabolism in mechanistic and observational studies [22–25]. Our results conform to another NHANES report in approximately 5000 US adults showing higher serum copper, selenium, and zinc to be associated with abnormal glucose metabolism [26]. In another cross-sectional study of 1197 adults recruited in Italy, dietary copper revealed a significant positive association with C-reactive protein [27]. Increased inflammation triggered by dietary factors, such as copper, can impair insulin signaling [28] and promote diabetes risks as observed in our nondiabetic controls. Free radical production is considered as one of the major mechanisms responsible for the adverse effects of copper and iron. Through the Fenton reaction, copper and iron can catalyze the generation of reactive oxygen species that can impair several physiological pathways including those related to insulin signaling [29]. In our study, these associations remained significant even in models adjusted for dietary patterns including major food groups, thereby indicating that copper can promote insulin resistance at habitual dietary intakes. While not significant, dietary intakes of copper and iron were higher in adults with T1D than nondiabetic controls in our cohort.

In contrast to dietary copper, dietary manganese revealed a protective association against higher HbA1c and promoted insulin sensitivity in our cohort. Dietary manganese intake has been inversely associated with the development of type 2 diabetes, as well as with biomarkers of inflammation in multiple prospective cohort studies [30–32]. Our findings of inverse association between dietary manganese and HbA1c in nondiabetic controls conform to these previous studies and suggest a role in the prevention of diabetes; associations were not as robust in adults with T1D suggesting manganese intake may not affect glycemic control during the established clinical course of the disease. Manganese is essential for the production and expression of oxidoreductases and manganese superoxide dismutase involved in the reduction of oxidative stress [33]. Manganese supplementation has also been shown to improve insulin secretion in mice [34], and also manganese-dependent superoxide dismutase has shown to protect against oxidative DNA damage in mitochondria in diabetes [35]. These functions may predict possible beneficial effects of manganese against diabetes.

Our observation of no association of dietary vitamins C and E with glycemic control conforms to the findings of meta-analyses which showed no beneficial effects of their supplementation in glycemic control and insulin resistance in adults with or without diabetes [15, 16, 36, 37]. In our cohort, vitamin C intakes were much above the dietary recommendations for adults (75-90 mg/day) [38], and vitamin E intakes were much lower than the recommended guidelines (<12 mg/day) [39]. These observations suggest the need to maintain habitual intakes of these vitamins at doses close to the recommended levels based on scientific evidence for optimal health effects.

Our study has some limitations as follows: Our cross-sectional analysis cannot address causality, and we did not measure serum/tissue levels of these antioxidant micronutrients which could shed greater light on these observed associations. Being limited to adults with established ASCVD and their matched nondiabetic controls, our findings cannot be generalized to other populations, namely, those with type 2 diabetes or gestational diabetes or younger adults with T1D but without ASCVD. Furthermore, in the current report, we analyzed dietary sources of these micronutrients and not those derived from dietary supplements, and their bioavailability that must be addressed in future studies. Finally, there is always the possibility of residual confounding in epidemiological studies that cannot be addressed in the analysis. Despite these limitations, the major strength of our study design is a well-characterized cohort of adults with T1D and their matched controls providing a clear comparison of associations between these two groups. Also, our significant findings remained robust in models extensively adjusted for major dietary patterns and food groups.

## 5. Conclusions

Dietary antioxidant nutrients, including vitamins and trace elements, play a major role in regulating antioxidant/oxidative stress pathways that have close implications in insulin signaling and glycemic control. Based on data collected from dietary intakes, and biochemical variables of glycated hemoglobin and insulin sensitivity, we examined associations of these factors in our cross-sectional observational study. Our findings reveal the importance of dietary copper and manganese in glycemic control in adults with type 1 diabetes, as well as in nondiabetic controls. Dietary copper may promote poor glycemic control, while manganese could be protective against hyperglycemia and promote insulin sensitivity. These nutrients and their food sources deserve attention in the nutrition therapy for diabetes and overall blood glucose control.

## Figures and Tables

**Table 1:** Baseline characteristics of participants.

Variables	Type 1 diabetes		Nondiabetic controls		*P* value
	*N* = 568		*N* = 689		
	Mean or *N*	SD or %^§^	Mean or *N*	SD or %^§^	
Age (years)	37	9	39	9	**<.0001**
Sex (female; *n*)	316	56	346	50	**0.03**
Diabetes duration (years)	23.5	8.9	—	—	—
HbA1c (%)	7.9	1.2	5.5	0.4	**<.0001**
HbA1c (met <7% goal)	113	20	N/A	N/A	N/A
Estimated insulin sensitivity (eIS)	6.4	3.0	15.2	8.5	**<.0001**
BMI (kg/m^2^)	26.2	4.3	26.2	5.0	0.92
LDL-C (mg/dL)	101	29	115	33	**<.0001**
Triacylglycerol (mg/dL)	93	54	132	103	**<.0001**
Dietary total calories (kcal/day)	1766	613	1822	619	0.11
Dietary carbohydrates (% kcal/day)	45	9	48	9	**<.0001**
Dietary fats (% kcal/day)	35	7	33	7	**<.0001**
Fruit, veggie, cereal, and meat pattern	0.06	1.35	-0.06	0.56	0.07
Baked desserts pattern	0.01	1.18	-0.006	0.85	0.77
Convenience foods and alcohol pattern	-0.04	0.86	0.03	1.11	0.22
Dietary vitamin C (mg/day)	121	70	118	71	0.36
Dietary vitamin E (mg/day)	5.5^#^	(4.1-7.5)^†^	5.5^#^	(4.3-7.5)^†^	0.53
Dietary iron (mg/day)	19.4	15.8	18.0	12.0	0.11
Dietary copper (mg/day)	1.9	0.94	1.5	0.92	0.33
Dietary zinc (mg/day)	18.6	14.6	17.0	14.1	0.05
Dietary selenium (*μ*g/day)	10.0	35.3	11.0	33.4	0.44
Dietary manganese (mg/day)	3.7	2.2	3.8	2.2	0.36
Dietary carotenoids (*μ*g/day)	9252^#^	(4162–11071) ^†^	8519^#^	(3996–10508) ^†^	0.23

^§^Column percentage. #Median. ^†^Interquartile range (Q1–Q3). *P* < 0.05 in bold font. N/A: not applicable.

**Table 2:** Cross-sectional associations of dietary antioxidant micronutrients with glycated hemoglobin (HbA1c) (*β* ± standard error).

Variable	Type 1 diabetes	*P* value	Nondiabetic controls	*P* value
Iron				
Model 1	0.005 ± 0.003	0.21	0.003 ± 0.001	0.82
Model 2	0.005 ± 0.004	0.18	0.005 ± 0.003	0.85
Model 3	0.004 ± 0.003	0.27	0.003 ± 0.001	0.82
Model 4	0.005 ± 0.003	0.21	0.004 ± 0.001	0.74
Copper				
Model 1	0.142 ± 0.103	0.17	0.054 ± 0.025	**0.03**
Model 2	0.111 ± 0.104	0.28	0.040 ± 0.025	0.11
Model 3	0.105 ± 0.101	0.30	0.040 ± 0.025	0.11
Model 4	0.168 ± 0.107	0.12	0.059 ± 0.025	**0.04**
Zinc				
Model 1	−0.001 ± 0.004	0.78	−0.003 ± 0.001	0.77
Model 2	−0.001 ± 0.003	0.90	−0.002 ± 0.001	0.70
Model 3	−0.001 ± 0.004	0.79	−0.002 ± 0.001	0.65
Model 4	−0.002 ± 0.004	0.62	−0.002 ± 0.001	0.81
Selenium				
Model 1	−0.001 ± 0.003	0.77	−0.001 ± 0.002	0.77
Model 2	−0.001 ± 0.002	0.72	−0.002 ± 0.003	0.78
Model 3	−0.001 ± 0.002	0.59	−0.004 ± 0.003	0.75
Model 4	−0.003 ± 0.002	0.84	−0.004 ± 0.003	0.77
Manganese				
Model 1	−0.065 ± 0.046	0.16	−0.031 ± 0.010	**0.007**
Model 2	−0.057 ± 0.045	0.22	−0.022 ± 0.011	**0.04**
Model 3	−0.035 ± 0.046	0.44	−0.023 ± 0.011	**0.04**
Model 4	−0.070 ± 0.048	0.13	−0.030 ± 0.010	**0.01**
Vitamin C				
Model 1	−0.001 ± 0.002	0.72	−0.002 ± 0.001	0.21
Model 2	−0.002 ± 0.001	0.76	−0.003 ± 0.001	0.20
Model 3	−0.001 ± 0.002	0.76	−0.002 ± 0.001	0.19
Model 4	−0.003 ± 0.002	0.69	−0.003 ± 0.001	0.22
Vitamin E				
Model 1	−0.002 ± 0.001	0.79	−0.002 ± 0.001	0.20
Model 2	−0.003 ± 0.004	0.89	−0.001 ± 0.001	0.24
Model 3	−0.001 ± 0.002	0.57	−0.002 ± 0.001	0.24
Model 4	−0.001 ± 0.002	0.77	−0.003 ± 0.001	0.20
Carotenoids				
Model 1	−0.001 ± 0.002	0.49	−0.002 ± 0.001	0.51
Model 2	−0.003 ± 0.002	0.35	−0.004 ± 0.002	0.49
Model 3	−0.003 ± 0.001	0.24	−0.003 ± 0.002	0.55
Model 4	−0.004 ± 0.001	0.48	−0.004 ± 0.002	0.62

^a^Model adjusted for age, sex, diabetes duration, and total calories for type 1 diabetes; age, sex, and total calories for nondiabetic controls. ^b^Model 1 + body mass index. ^c^Model 1 + plasma LDL cholesterol and triglycerides. ^d^Model 1 + principal component analysis-derived dietary patterns including all food groups (“fruits, veggies, meats, cereal pattern,” “baked desserts pattern,” and “convenience foods and alcohol pattern”). *P* ≤ 0.05 in bold font.

**Table 3:** Cross-sectional associations of dietary antioxidant micronutrients with estimated insulin sensitivity (*β* ± standard error).

Variable	Type 1 diabetes	*P* value	Nondiabetic controls	*P* value
Iron				
Model 1^a^	−0.012 ± 0.006	0.13	0.002 ± 0.001	0.73
Model 2^b^	−0.021 ± 0.006	0.11	0.002 ± 0.005	0.71
Model 3^c^	−0.013 ± 0.006	0.24	0.002 ± 0.006	0.86
Model 4^d^	−0.017 ± 0.007	0.21	0.007 ± 0.006	0.90
Copper				
Model 1	−0.243 ± 0.178	0.17	−0.850 ± 0.449	0.06
Model 2	−0.018 ± 0.058	0.58	−0.789 ± 0.386	**0.05**
Model 3	−0.205 ± 0.169	0.22	−0.688 ± 0.100	**0.04**
Model 4	−0.286 ± 0.184	0.12	−0.614 ± 0.103	**0.04**
Zinc				
Model 1	0.007 ± 0.006	0.31	−0.008 ± 0.005	0.87
Model 2	0.004 ± 0.005	0.49	−0.004 ± 0.005	0.92
Model 3	0.006 ± 0.005	0.35	−0.006 ± 0.005	0.91
Model 4	0.007 ± 0.005	0.32	−0.007 ± 0.005	0.97
Selenium				
Model 1	−0.001 ± 0.002	0.79	−0.002 ± 0.001	0.44
Model 2	−0.001 ± 0.002	0.99	−0.016 ± 0.005	0.33
Model 3	−0.003 ± 0.002	0.99	−0.005 ± 0.001	0.42
Model 4	−0.007 ± 0.003	0.97	−0.003 ± 0.001	0.48
Manganese				
Model 1	0.217 ± 0.080	**0.03**	0.544 ± 0.204	**0.008**
Model 2	0.104 ± 0.076	0.17	0.389 ± 0.182	**0.03**
Model 3	0.110 ± 0.077	0.15	0.367 ± 0.146	**0.03**
Model 4	0.167 ± 0.082	0.06	0.319 ± 0.148	**0.02**
Vitamin C				
Model 1	0.004 ± 0.002	0.12	0.005 ± 0.002	0.91
Model 2	0.003 ± 0.002	0.20	0.002 ± 0.001	0.87
Model 3	0.003 ± 0.002	0.12	0.002 ± 0.001	0.97
Model 4	0.003 ± 0.001	0.13	0.003 ± 0.001	0.96
Vitamin E				
Model 1	0.004 ± 0.001	0.64	0.002 ± 0.001	0.18
Model 2	0.003 ± 0.002	0.47	0.003 ± 0.001	0.32
Model 3	0.003 ± 0.002	0.99	0.005 ± 0.003	0.15
Model 4	0.002 ± 0.001	0.54	0.005 ± 0.003	0.18
Carotenoids				
Model 1	0.002 ± 0.001	0.52	0.004 ± 0.001	0.64
Model 2	0.002 ± 0.001	0.94	0.004 ± 0.001	0.55
Model 3	0.002 ± 0.001	0.97	0.003 ± 0.001	0.60
Model 4	0.003 ± 0.001	0.61	0.003 ± 0.001	0.46

^a^Model adjusted for age, sex, diabetes duration, and total calories for type 1 diabetes; age, sex, and total calories for nondiabetic controls. ^b^Model 1 + body mass index. ^c^Model 1 + plasma LDL cholesterol and triglycerides. ^d^Model 1 + principal component analysis-derived dietary patterns including all food groups (“fruits, veggies, meats, cereal pattern,” “baked desserts pattern,” and “convenience foods and alcohol pattern”). *P* ≤ 0.05 in bold font.

## Data Availability

The datasets analyzed in the current study are not publicly available due to ethical reasons and because our participants only gave their consent for the use of their data by the original team of investigators.
